# The Role of Phyllosphere Microbes and Viruses in Biocontrol of Pathogenic Fungi

**DOI:** 10.1111/1751-7915.70251

**Published:** 2025-10-10

**Authors:** Li Bi, Zahra F. Islam, Lok‐Hang Chan, Hang‐Wei Hu

**Affiliations:** ^1^ School of Agriculture, Food and Ecosystem Sciences, Faculty of Science The University of Melbourne Parkville Victoria Australia; ^2^ ARC Research Hub for Smart Fertilisers The University of Melbourne Parkville Victoria Australia

**Keywords:** fungal viruses, pathogen suppression mechanisms, pathogenic fungi, phyllosphere

## Abstract

The phyllosphere, the aerial surfaces of plants, represents a primary entry point for airborne fungal pathogens, posing a critical challenge to plant health and productivity. The phyllosphere hosts diverse microbial communities that play a pivotal role in suppressing foliar pathogens through complex ecological interactions. In this mini review, we synthesise recent advances in understanding how phyllosphere microbial diversity contributes to fungal pathogen suppression through multiple ecological mechanisms, including resource competition, secretion of antifungal metabolites, contact‐dependent killing and activation of host immune responses. We highlight emerging evidence on the role of viruses in controlling fungal pathogens and propose a conceptual framework based on virus‐mediated strategies for fungal disease control. We emphasise that better mechanistic understanding of plant–fungus–microbiota interactions is critical to developing sustainable and microbiota‐based approaches for plant resilience enhancement and global food security within a One Health framework.

## Graphical abstract

Foliar microbiota employ multiple ecological strategies to inhibit fungal pathogens in the phyllosphere, including resource competition, production of antifungal metabolites, contact‐dependent killing, modulation of plant innate immunity and mycovirus‐mediated suppression.

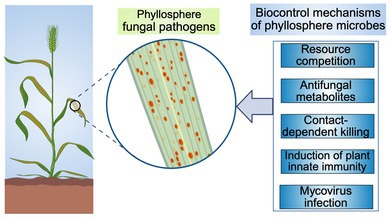

## 1 Introduction

Fungal pathogens are among the most devastating agents of plant disease and pose a significant threat to global agricultural production and food security (Fones et al. 2020). Fungal infections are estimated to destroy over 125 million tonnes of major crop production, such as wheat, rice, maize, soybean and potato, resulting in an annual cost of US$60 billion to the global agriculture industry (Fisher et al. 2012; Ristaino et al. 2021). Fungal pathogens are projected to increasingly impact crop yields under future warming scenarios (Delgado‐Baquerizo et al. 2020). These pathogens infect various plant organs, while foliar tissues are particularly susceptible due to their direct exposure to airborne spores and fluctuating environmental conditions (Vorholt 2012). The phyllosphere, comprising the aerial surfaces of plants, including leaves, stems, flowers and fruits, is frequently colonised by diverse fungal pathogens that cause major foliar diseases such as rusts, mildews and blights. A well‐known example is the epidemic of wheat stem rust caused by *Puccinia graminis f*. sp. *tritici*, a fungal pathogen that has repeatedly threatened global wheat production and continues to cause major losses despite ongoing control efforts (Singh et al. 2011). The persistence and severe ecological consequences of foliar fungal pathogens highlight the importance of developing effective approaches for long‐term disease management.

**FIGURE 1:**
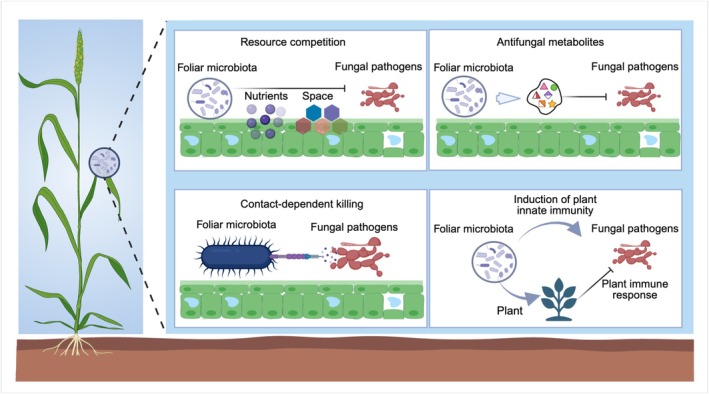
Ecological mechanisms by which microbes suppress fungal pathogens in the phyllosphere. Foliar microbiota employ multiple ecological strategies to inhibit fungal pathogens in the phyllosphere. These include: (i) resource competition, where microbiota outcompete pathogens for limited spaces and nutrients; (ii) production of antifungal metabolites, which disrupt fungal development and viability; (iii) contact‐dependent killing, which directly inhibits fungal cells through intercellular contact; and (iv) modulation of plant innate immunity, in which commensal microbes induce host defence responses to prevent pathogen invasion.

Historically, fungal‐disease management has relied heavily on chemical fungicides, fungal‐resistant crop varieties and certain agricultural practices (e.g., fallow and soil fumigation). However, these strategies face increasing challenges due to short‐term effectiveness, the emergence of fungicide‐resistant strains, environmental contamination, and growing societal and regulatory pressure for more sustainable solutions (Grimmer et al. 2015; Fisher et al. 2018). As agricultural systems move towards more sustainable and ecologically grounded practices, there is growing interest in the use of plant‐associated microbes, their interactions and metabolites as a natural means of disease suppression (Peixoto et al. 2022; Jian et al. 2024). The phyllosphere harbours diverse microbial communities, including bacteria, fungi and viruses, that have been increasingly shown to suppress fungal pathogens through multiple ecological mechanisms (Zhan et al. 2022; Figure 1). Although several mechanistic pathways have begun to emerge, the molecular mechanisms through which phyllosphere microbiota suppress fungal pathogens remain insufficiently understood. Further in‐depth mechanistic investigations are needed to improve our understanding of plant–microbiome interactions, thereby providing novel alternatives to conventional disease resistance strategies.

In this article, we synthesise recent advances in understanding the ecological roles of phyllosphere microbes in fungal foliar disease resistance, including resource competition, production of antifungal compounds, contact‐dependent killing and induction of host immune responses. We further highlight emerging insights into virus‐based strategies for fungal pathogen control and propose a conceptual framework to guide future exploration of viral contributions to plant protection. Together, this review aims to enhance our understanding of plant–microbe–environment interactions and foster the integration of phyllosphere microbiome research into sustainable disease management.

## 2 Foliar Pathogenic Fungi Suppression Mechanisms

### 2.1 Resource Competition

In the phyllosphere, fungal pathogens often initiate infection through specific microsites, such as stomata, trichome bases and epidermal grooves. These sites are limited in number and represent valuable ecological niches (Remus‐Emsermann and Schlechter 2018). Non‐pathogenic microbes that colonise the leaf surface early can occupy these microsites and form biofilms, thereby physically blocking pathogen access and establishment. Such spatial exclusion is a well‐recognised mechanism of microbial‐mediated disease suppression (Figure 1; Chepsergon and Moleleki 2023; Roussin‐Léveillée et al. 2024). Nutrient competition among microbes is also considered an important mechanism for pathogen suppression. Iron, an essential but poorly available micronutrient, is a prime target of microbial competition (Liu et al. 2021). In the rhizosphere, some bacteria secrete siderophores (high‐affinity iron‐chelating compounds) to scavenge iron exclusively for their own use, depriving pathogens of this vital resource. A previous study demonstrated that siderophore‐producing rhizobacteria effectively inhibited *Ralstonia solanacearum* by limiting iron availability (Gu et al. 2020). Similar iron‐driven competitive interactions may occur in the phyllosphere, as suggested by studies of microbial dynamics on citrus leaves infected with *Diaporthe citri*, a fungal pathogen causing melanose disease worldwide (Li et al. 2022). While siderophore‐mediated competition is a well‐established mechanism for bacterial pathogen control in the rhizosphere (Qin et al. 2019), direct experimental evidence for fungal pathogen suppression in the phyllosphere remains limited largely due to the nutrient‐poor conditions of leaf surfaces and strong environmental fluctuations that hinder the stability and detection of such interactions. Future studies should employ genetic manipulation of siderophore biosynthetic pathways and functional assays with synthetic microbial communities under field‐relevant conditions to establish causal links between siderophore competition and foliar fungal pathogen suppression. Competition for limited nutrients (e.g., carbon sources and trace elements) may also contribute to the suppression of foliar fungal pathogens (Gouka et al. 2022; Schäfer et al. 2023). Moreover, the availability of leaf surface moisture is critical for microbial colonisation and pathogen infection. For example, certain pathogens actively manipulate host physiology to establish localised aqueous niches that are essential for their virulence (Xin et al. 2016).

### 2.2 Secretion of Antifungal Metabolites

Specialised metabolites produced by phyllosphere microbes represent a pivotal ecological mechanism for suppressing foliar fungal pathogens (Figure 1). These compounds, including both volatile and non‐volatile molecules, can interfere with multiple key stages of fungal development, such as mycelial growth, appressorium formation, sclerotia production and hyphal expansion (Xu et al. 2021). Recent studies have illuminated the biochemical diversity and ecological relevance of this mechanism. For instance, *Aspergillus cvjetkovicii*, an indigenous fungal species from the asymptomatic rice phyllosphere, was found to secrete small molecules such as 2(3H)‐benzofuranone and azulene, which inhibited different key developmental stages of *Magnaporthe oryzae*, a devastating fungal pathogen of rice (Fan et al. 2019). Field trials further demonstrated that *A. cvjetkovicii* could significantly reduce *M. oryzae* epidemics by up to 68.5% (Fan et al. 2019). A key antifungal molecule (2,4‐di‐tert‐butylphenol) derived from *A. cvjetkovicii* demonstrated protective effects against *Rhizoctonia solani* infection in diverse crops and its broad‐spectrum inhibitory activity against other fungal pathogens (Fan et al. 2024). In addition, some bacterial members produced secondary metabolites that directly affected the activity of fungal proteins, which suppressed the growth and virulence of *Fusarium graminearum*, thereby compromising its pathogenicity (Chen et al. 2018).

### 2.3 Contact‐Dependent Killing

To outcompete neighbouring microbes, certain bacteria employ contact‐dependent killing strategies involving specialised secretion systems, such as type IV (T4SS), type VI (T6SS) and type VII secretion systems (T7SS), to directly deliver toxic effectors into rival cells. This mechanism typically relies on intimate cell‐to‐cell contact and has recently gained attention as a potent means of microbial interference and pathogen suppression (Figure 1; Shao et al. 2024). For instance, *Rhizobium* Leaf202 has been reported to employ T6SS to suppress bacterial pathogens in the phyllosphere (Vogel et al. 2021). Recent studies showed that antibiotic‐deficient bacterial mutants of *Lysobacter enzymogenes* and *Pseudomonas fluorescens* effectively inhibited filamentous fungal pathogens through T6SS‐mediated intercellular contact (Lin et al. 2025). The role of T6SS in enhancing plant protection was further assessed through leaf inoculation assays, where co‐application of these bacterial strains and fungal pathogens on soybean leaves significantly reduced fungal colonisation. In contrast, the T6SS‐deficient mutants showed markedly diminished antagonistic activity. Notably, this T6SS‐mediated contact‐dependent inhibition was also observed in natural soil microbiomes (Lin et al. 2025), highlighting its ecological relevance and potential as an antibiotics‐independent strategy for engineering beneficial microbial consortia against plant fungal diseases.

### 2.4 Induction of Plant Innate Immunity

Pathogenic microbes have long been recognised to elicit plant immune responses, which contribute to disease suppression (Chisholm et al. 2006). Pathogen‐induced immunity is primarily mediated via recognition of microbe‐associated molecular patterns (MAMPs), ferroptosis‐related signalling or microbial metabolites, which can initiate either local or systemic resistance (Du et al. 2025). Growing evidence reveals that non‐pathogenic or beneficial microbial members can also activate plant immune responses through their intricate interactions with plants, thereby contributing to foliar fungal pathogen suppression (Figure 1; Van Wees et al. 2008; Du et al. 2025). For instance, *Protomyces arabidopsidicola*, a commensal fungal species isolated from the *Arabidopsis* phyllosphere, was shown to enhance host resistance against *Botrytis cinerea* by activating the mitogen‐activated protein kinase cascades and multiple defence hormone pathways (Wang et al. 2022). Similarly, a synthetic eubiotic community comprising 48 leaf‐derived bacterial strains restored immune competence in *Arabidopsis* and conferred resistance to *B. cinerea* through reactivation of pattern‐triggered immunity (Paasch et al. 2023). However, microbiota‐mediated immune priming may involve trade‐offs, as enhanced defence capacity can come at the expense of plant growth due to growth‐defence allocation (He et al. 2022; Sohrabi et al. 2023). Many commensals may also facilitate disease progression by suppressing host immunity, acting as so‐called ‘pathogen helpers’ (Yu et al. 2019; Du et al. 2025). Moreover, some beneficial microbes express MAMPs that resemble those of pathogens, posing a challenge for the plant immune system to distinguish mutualists from invaders (Teixeira et al. 2019). These findings emphasise the immunological complexity of plant–microbiota interactions. Although promising, the application of microbiota‐mediated immune priming in managing foliar fungal diseases remains at an early stage, and a deeper mechanistic understanding is essential for translating these interactions into reliable biocontrol strategies.

## 3 Embedding Viruses for Fungal Pathogen Suppression

### 3.1 Introduction to Viruses as Biocontrol Agents

Over the past decades, viruses, as the most abundant entities in the world, have garnered increasing attention across soil and plant ecosystems (Bi et al. 2024; Carreira et al. 2024). Viral lifestyle strategies, particularly the lytic cycle, enable viruses to selectively lyse target hosts and thereby alter host cell densities and microbial population structure (Kuzyakov and Mason‐Jones 2018). Based on their host‐specific lytic capacity, viruses, as part of the phyllosphere microbiota, can act as precision biocontrol agents against pathogens. This virus‐mediated biocontrol offers an alternative to traditional microbial biocontrol strategies (Brives and Pourraz 2020; Wagemans et al. 2022). In particular, bacteriophages infecting bacteria have shown promising effects on bacterial plant disease suppression (Wang, Tang, et al. 2024). For instance, phage application significantly reduced the incidence of tomato bacterial wilt caused by *R. solanacearum* (Wang et al. 2019; Wang, Wang, et al. 2024) and the abundance of *Pseudomonas syringae* on cherry leaves in both greenhouse and field trials (Papp‐Rupar et al. 2025), and inhibited the growth of *Xanthomonas* and the associated disease symptoms on rice leaves (Liu et al. 2023). Despite the substantial economic burden imposed by fungal pathogens, virus‐based biocontrol strategies against fungal diseases remain comparatively underexplored relative to phage‐based approaches in agriculture.

### 3.2 Fungal Viruses and Their Potential in Biocontrol

Fungal viruses (mycoviruses) are typically obligate intracellular parasites of fungi, comprising either single‐stranded RNA, double‐stranded RNA or single‐stranded DNA genomes. These genomes can be monopartite or multipartite, and the viruses may exist in naked or encapsidated forms (Wagemans et al. 2022). Unlike phages, most mycoviruses lack an extracellular transmission phase and are primarily transmitted vertically through sporulation or horizontally via hyphal anastomosis (Xie and Jiang 2024). While many mycoviruses cause latent infections with no observable phenotypic changes, a subset known as hypovirulence‐associated mycoviruses (HAVs) is capable of attenuating key fungal traits (e.g., virulence), thus reducing the pathogenicity of their fungal hosts (Kondo et al. 2022). These HAVs have attracted increasing interest for their potential application in virus‐based biocontrol of plant fungal diseases (Xie and Jiang 2024).

The earliest and most successful documented use of viruses to control fungal pathogens is the application of the mycovirus *Cryphonectria hypovirus 1* (*CHV1*), a representative HAV, to suppress the chestnut blight fungus *Cryphonectria parasitica* (Anagnostakis 1982). However, subsequent field applications of mycoviruses in chestnut blight have shown inconsistent results across different regions, particularly between Europe and North America. One major limitation is the vegetative incompatibility among fungal strains, a non‐self allorecognition system, which restricts hyphal fusion and thereby limits the horizontal transmission of HAVs via anastomosis (Turina and Rostagno 2007; Xie and Jiang 2024). Since HAVs typically lack extracellular infection phases and depend entirely on intracellular spread via fungal anastomosis, their field performance is tightly linked to host population compatibility. A recent study found that oilseed rape infection by *Sclerotinia sclerotiorum* triggers proline accumulation in host tissue, which weakens the fungal non‐self recognition response and facilitates HAV spread even between incompatible strains (Hai et al. 2024). Field trials further showed that co‐application of hypovirulent *S. sclerotiorum* strains carrying HAVs with exogenous proline conferred substantial protection to plants against virulent *S. sclerotiorum*. This highlights a novel plant‐mediated strategy that boosts viral biocontrol efficacy by modulating fungal innate barriers and enhancing HAV dissemination in natural settings.

### 3.3 A New Framework for Virus‐Based Biocontrol of Foliar Fungal Pathogens

**FIGURE 2:**
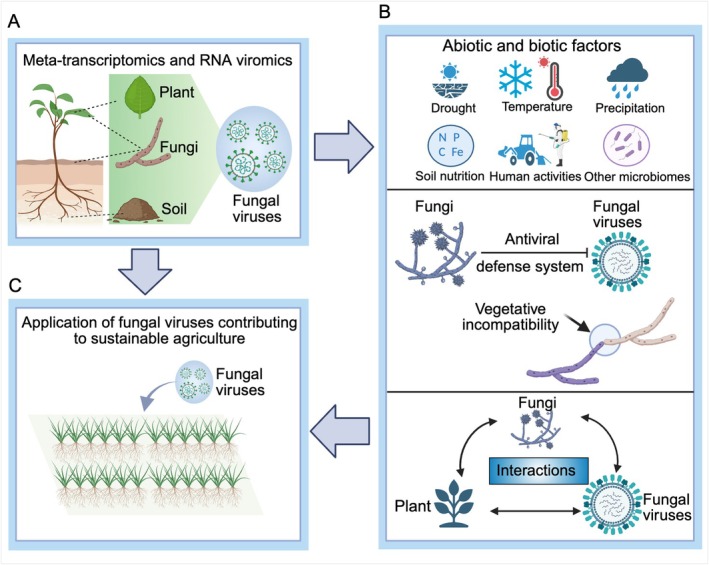
Conceptual framework for virus‐based control of fungal pathogens in the phyllosphere. (A) Uncovering the diversity of fungal viruses associated with foliar fungal pathogens across different ecological compartments, including cultured fungi, plants and soil using multiple metagenomic methods. (B, C) An integrative understanding of abiotic and biotic drivers, fungal antiviral defences and tripartite interactions among viruses, fungi and host plants will inform the development of mycovirus‐based biocontrol strategies against foliar fungal pathogens, offering a promising route to enhance plant resilience and support sustainable crop production.

Here, we propose a new framework to facilitate the development of virus‐based strategies for controlling foliar pathogenic fungi. Firstly, it is imperative to construct a global atlas of their associated mycoviruses, encompassing their genomic features, host ranges and ecological distributions. Advanced high‐throughput approaches, such as meta‐transcriptomics and RNA viromics, offer powerful platforms for capturing actively replicating or low‐abundance viral taxa, as well as inferring host–virus interactions in situ (Chen et al. 2022; Hillary et al. 2022; Yan et al. 2025). For the comprehensive discovery and characterisation of these mycoviruses, all habitats including plants, especially the phyllosphere, soils and cultured foliar pathogenic fungi, should be considered (Figure 2). Moreover, the multifaceted complexity of agroecosystems (e.g., fluctuating climatic regimes, heterogeneous soil properties, human activities and intricate microbial networks) can influence viral persistence, host compatibility and horizontal transmission dynamics. These ecological variables may potentially hinder the predictable deployment of mycoviruses for fungal disease control. Future efforts should prioritise disentangling these ecological drivers through integrated omics, long‐term field surveys and predictive ecological modelling.

The practical application of mycoviruses as biocontrol agents remains challenging. Fungi, like other eukaryotes, possess multiple antiviral defence strategies (Duan and Liu 2025). These mechanisms include vegetative incompatibility, RNA interference, symptom alleviation pathways and RNA editing (Duan and Liu 2025), which can significantly restrict viral replication, accumulation and horizontal transmission across fungal strains. Among these, vegetative incompatibility has received particular attention in the context of mycovirus‐based biocontrol (Figure 2). Beyond plant‐derived signals like proline that can suppress fungal non‐self recognition (Hai et al. 2024), several additional strategies show promise in overcoming vegetative incompatibility barriers (Xie and Jiang 2024). For example, targeted disruption of *vic* or *het* loci has enabled the construction of engineered ‘super donor’ strains in *C. parasitica* , greatly enhancing inter‐strain viral dissemination (Zhang and Nuss 2016). Some mycoviruses themselves, such as SsHADV1, can avoid incompatibility barriers and directly infect S. *sclerotiorum* hyphae extracellularly, protecting plants against *Sclerotinia*‐induced diseases (Yu et al. 2013), while others, such as SsMYRV4, can suppress the vegetative incompatibility response to facilitate heterologous viral transfer (Galli et al. 2025). Chemical interventions, including zinc‐based compounds, have also been shown to modulate programmed cell death pathways, providing additional means to promote horizontal spread (Ikeda et al. 2013; Xie and Jiang 2024).

Finally, advancing the application of mycoviruses as biocontrol agents in the phyllosphere also calls for a deeper integration of the complex tripartite interactions among plants, foliar fungal pathogens and their associated viruses (Figure 2). Rather than merely acting as intracellular inhibitors of fungal virulence, certain mycoviruses function as ecological modulators capable of reshaping host‐pathogen‐plant relationships. An example is the hypovirulence‐associated DNA virus SsHADV1, which not only attenuates the pathogenicity of *S. sclerotiorum* but also enables the fungus to adopt an endophytic lifestyle within rapeseed (Zhang et al. 2020). Once transformed, the fungus loses its ability to cause disease and instead promotes plant growth and immunity, effectively converting a necrotrophic pathogen into a beneficial symbiont. This virus‐mediated shift illustrates the potential for mycoviruses to serve as live biocontrol agents, or ‘plant vaccines’, capable of colonising host tissues while enhancing resistance at the whole‐plant level (Liu et al. 2022; Xie and Jiang 2024). In addition, recent research found that Diaporthe sojae circular DNA virus 1 (DsCDV1) phylogenetically situated between fungal and plant viral clades can bypass the fungal host altogether and systemically infect plants via a movement protein that facilitates cell‐to‐cell spread (Wang, Kotta‐Loizou, et al. 2024). Notably, DsCDV1 confers broad‐spectrum resistance against diverse fungal pathogens, positioning it as a trans‐kingdom viral agent that bridges fungal and plant ecological niches. Taken together, these findings highlight the need to move beyond a pathogen‐centric view and instead recognise mycoviruses as active participants in plant–microbe interaction networks. A mechanistic understanding of these virus‐mediated symbioses could inform the development of field‐deployable strategies for next‐generation biocontrol strategies that harness naturally evolved viral functions to stabilise plant health across dynamic agroecosystems.

## 4 Conclusion

Advances in microbiome ecology have highlighted the critical role of microbial members in suppressing pathogens through mechanisms such as niche competition, antagonistic metabolites, contact‐dependent killing and activation of host immune responses. The discovery of hypovirulence‐associated mycoviruses and their ability to convert pathogenic fungi into endophytes or attenuated strains further expands the biocontrol toolbox. However, translating these discoveries into reliable and effective field applications remains a major challenge, hindered by the fragmented understanding of microbiota–fungal pathogen–plant interactions and the inherent ecological complexity of the phyllosphere. Future research should therefore extend beyond controlled models to embrace integrative and field‐based long‐term studies that better reflect ecological complexity, enabling the development of ecological and predictive models to forecast disease suppression outcomes and guide the design of microbial consortia for sustainable crop protection. Meanwhile, the proposed virus‐based framework will further advance field‐deployable biocontrol approaches. Together, these efforts will help bridge the gap between mechanistic insights and applied outcomes, fostering informed practices that are resilient to environmental change and aligned with sustainable agriculture.

## Author Contributions

**Li Bi:** conceptualization, visualization, writing – original draft, writing – review and editing. **Zahra F. Islam:** writing – original draft, writing – review and editing. **Lok‐Hang Chan:** writing – original draft, writing – review and editing. **Hang‐Wei Hu:** conceptualization, funding acquisition, project administration, supervision, writing – original draft, writing – review and editing.

## Conflicts of Interest

The authors declare no conflicts of interest.

## Data Availability

Data sharing is not applicable to this article as no new data were created or analysed in this study.
